# Vulnerability to physical inactivity: evidence of content validity and response processes

**DOI:** 10.1590/0034-7167-2022-0563

**Published:** 2023-11-13

**Authors:** Déborah Santana Pereira, Raquel Sampaio Florêncio, Virna Ribeiro Feitosa Cestari, Thereza Maria Magalhães Moreira

**Affiliations:** I Instituto Federal de Educação, Ciência e Tecnologia do Ceará. Juazeiro do Norte, Ceará, Brazil; II Universidade Estadual do Ceará. Fortaleza, Ceará, Brazil

**Keywords:** Health Vulnerability, Sedentary Behavior, Adult, Psychometrics, Validation Study, Vulnerabilidad en Salud, Conducta Sedentaria, Adulto, Psicometría, Estudio de Validación, Vulnerabilidade em Saúde, Comportamento Sedentário, Adulto, Psicometria, Estudos de Validação

## Abstract

**Objective:**

To analyze content validity evidence and response processes of a bank of items for measuring vulnerability to physical inactivity in adults.

**Method:**

Methodological study, with 13 specialists and 46 representatives of the target population. The Content Validity Index (CVI) and binomial test were calculated; data obtained through validity based on response processes were collected through interviews.

**Results:**

Of the 105 constructed items, 16 were excluded (CVI<0.78); 89 items showed agreement <80% in the psychometric criteria, being modified. Of the 101 items that remained (CVI>0.78), 34 were changed and 4 were deleted after evaluating the evidence of response processes. In the end, 97 items remained, with a global CVI of 0.92, organized into two dimensions: Subject (CVI=0.91) and Social (CVI=0.94).

**Conclusion:**

The items presented adequate parameters and evidence of validity; and can subsidize the construction of instruments that consider the subject’s and social vulnerability in understanding physical inactivity.

## RESUMO

**Objetivo:**

Analisar evidências de validade de conteúdo e processos de resposta de um banco de itens para mensuração da vulnerabilidade à inatividade física de adultos.

**Método:**

Estudo metodológico, com 13 especialistas e 46 representantes da população-alvo. Calculou-se o Índice de Validade de Conteúdo (IVC) e teste binomial; os dados obtidos pela validade baseada nos processos de respostas foram coletados mediante entrevistas.

**Resultados:**

Dos 105 itens construídos, 16 foram excluídos (IVC<0,78); 89 itens apresentaram concordância <80% nos critérios psicométricos, sendo modificados. Dos 101 itens que permaneceram (IVC>0,78), 34 foram alterados e 4 eliminados após avaliação das evidências dos processos de resposta. Ao final, permaneceram 97 itens, com IVC global de 0,92, organizados em duas dimensões: Sujeito (IVC=0,91) e Social (IVC=0,94).

**Conclusão:**

Os itens apresentaram parâmetros e evidências de validade adequados; e podem subsidiar a construção de instrumentos que consideram a vulnerabilidade do sujeito e social na compreensão da inatividade física.

## RESUMEN

**Objetivo:**

Analizar evidencias de validez de contenido y procesos de respuesta de un banco de ítems para medición de vulnerabilidad al sedentarismo de adultos.

**Método:**

Estudio metodológico, con 13 especialistas y 46 representantes de la población objetivo. Calculado Índice de Validez de Contenido (IVC) y prueba binomial; datos obtenidos por validez basada en procesos de respuestas recolectados mediante entrevistas.

**Resultados:**

De los 105 ítems construidos, 16 fueron excluidos (IVC<0,78); 89 ítems presentaron concordancia <80% en los criterios psicométricos, siendo modificados. De los 101 ítems que permanecieron (IVC>0,78), 34 fueron alterados y 4 eliminados pos evaluación de evidencias de procesos de respuesta. Al fin, permanecieron 97 ítems, con IVC global de 0,92, organizados en dos dimensiones: Sujeto (IVC=0,91) y Social (IVC=0,94).

**Conclusión:**

Los ítems presentaron parámetros y evidencias de validez adecuados; y pueden subsidiar la construcción de instrumentos que consideran la vulnerabilidad del sujeto y social en la comprensión del sedentarismo.

## INTRODUCTION

One of the essential health elements for the healthy development and quality of life of people is physical activity^(1)^, however, there is a high prevalence of physical inactivity, defined here as the condition of not meeting the recommendations for moderate to vigorous intensity physical activity^(2, 3)^. Due to its individual and social impact^(4, 5)^, efforts have been made to include the regular practice of physical activities in the daily lives of people around the world^(6)^ and in Brazil^(7)^.

Without ignoring individual aspects, such as beliefs, affections and feelings, social inequalities have been identified as strong influencers on adherence to physical activity, especially during leisure. Some socially and economically less privileged groups tend to be at a notable disadvantage in terms of possibilities and options for regular practice of physical activities^(8, 9)^. Thus, analyzing the main vulnerability factors that contribute to the outcome “physical inactivity” is a promising means for planning and implementing strategies to promote physical activity for the health of different populations^(10, 11)^.

The vulnerability approach has been used to discover how the interaction between individual aspects and cultural and social dynamics can increase people’s susceptibility to harm, threats or dangers^(10)^. In this sense, vulnerability to physical inactivity is understood as a condition generated through the interaction between elements of multiple dynamic relationships of the subject in their social context, which produces greater precariousness and exposure to unfavorable factors of physical activity for health, without means of coping^(12)^.

The Subject Dimension of vulnerability to physical inactivity shows how much personal aspects can increase the chances of physical inactivity, understood here as the subject as a producer and as a product of the power relations existing in society. The Social Dimension reflects the understanding of physical inactivity based on the subject’s multiple collective and contextual elements^(11, 12)^.

Currently, there are measurement instruments that assess aspects of physical (in)activity, such as the level of physical activity^(13, 14)^ and perception of barriers to practice^(15)^, however, they do not understand this construct in its entirety and complexity. Most evaluate only a few elements from an individual perspective, without considering the subjectivities and the questioning of health inequalities that involve some population groups. Thus, there is still a need for instruments, with evidence of validity, that enable sufficient and reliable data to understand contemporary, individual, collective, and contextual attributes, in an articulated way — for example, those understood from the perspective of subject and social vulnerabilities.

From this perspective, a bank of items, that is, a set of evaluative questions, with evidence of the validity of its content by specialists and an approach to aspects of vulnerability in the context of physical inactivity, enables the development of measurement instruments, which help in the understanding the level of susceptibility to physical inactivity and identifying personal and social factors that influence this outcome. With these results, specific health intervention strategies can be proposed, according to the identified need.

## OBJECTIVE

To analyze content validity evidence and response processes of a bank of items for measuring vulnerability to physical inactivity in adults.

## METHODS

### Ethical aspects

This study was approved by the Ethics Committee of the State University of Ceará and developed according to the ethical precepts contained in Resolution 466/2012 of the National Health Council of Brazil^(16)^. Free and Informed Consent was obtained from all individuals involved in the study, in writing (target population) and online (experts).

### Study design, period, and place

Methodological study, based on the first of the three poles of psychometrics for the development and validation of instruments (theoretical, empirical and analytical)^(17)^. Therefore, it refers to the theoretical pole, which includes the phases of construction and evidence of content validity and response processes: 1) construction of the item bank based on the constitutive and operational definitions of the studied construct; 2) search for sources of evidence of validity of the content of the items according to experts; 3) response processes.

Content validation took place in a virtual environment, covering seven states in Brazil. The validity based on the response processes took place in September 2021, in the city of Juazeiro do Norte, located in the central area of the Metropolitan Region of Cariri, in the south of the state of Ceará.

### Population or sample; inclusion and exclusion criteria

For the content validation stage, 25 specialists were invited^(17)^, with academic training, professional activity and scientific production and consistent with the subject studied, in addition to knowledge about methodological studies. For the phase of evidence based on the response processes, the target population of this study was considered, namely adults, aged between 18 and 59 years, as indicated in public policies to promote physical activity at the national level^(7)^. Thus, a representative sample of all strata of this population was selected: 46 adults of both genders, residing in the collection site for at least six months. Institutionalized adults, unable to communicate and who withdrew from participating in the research were excluded.

### Study protocol

The definition of the construct “Vulnerability to Physical Inactivity” and the elucidation of its dimensionality, constitutive elements, as well as constitutive and operational definitions of its markers were given through a literature review, critical reflection of the authors and evidence of validity of its content by specialists^(12)^. To define the behaviors and attitudes through which this construct is expressed, an item bank was created, based on psychometric criteria recommended by Pasquali^(17)^: relevance, precision, objectivity, simplicity, clarity, variety, modality, typicality, credibility , breadth and balance.

In content validation, the following criteria were used to select specialists: academic background, knowledge about methodological studies, scientific production, and professional performance. The selection took place through consultation with the Lattes Platform and the national database of the Coordination for the Improvement of Higher Education Personnel (CAPES), and the specialists were contacted and invited to participate in the study by email, with the indication of other professionals also being required with similar profile.

After acceptance, an email was sent with the Free and Informed Consent Form (FICF), an evaluation instrument prepared by the author and instructions for completing it. We chose to develop this instrument because of the quantity and specificity of the items to be evaluated and the established psychometric criteria. It is a spreadsheet created in the Microsoft Excel® program, with the items and criteria considered in the evaluation, in addition to spaces for inclusion of possible specialist suggestions.

The 25 experts invited had 15 to 20 days to judge the relevance and pertinence of the items in relation to the studied construct. This evaluation focused on the objectivity, simplicity, clarity, precision, and relevance of the items, analyzed on a four-point ordinal scale: 1) not indicative; 2) not very indicative; 3) indicative; 4) very indicative. Then, the data were analyzed by calculating the Content Validity Index (CVI) and the agreement on the psychometric criteria^(17)^.

Next, an attempt was made to analyze the understanding of the items for all segments of the population for which they were developed. In this way, the response processes were performed, as evidence that gathers arguments denoting the consistencies between the responses and the processes for which the proposed tasks are established^(18)^. The set of items was applied in September 2021, to people from the lowest to highest levels of education (considering years of study and grade) of the target population.

The 46 adults were randomly selected and invited to participate in the research voluntarily. Items were evaluated for understand-ability of words, terms, meaning and format. The evaluation of the items in the response processes was carried out in the form of an individual interview, and the researcher observed the participants’ reaction to each of the items, identifying difficulties and weaknesses. Each suggestion given regarding the elimination of an item or the best way of presenting it was recorded in writing for later analysis and amendment.

It should be noted that social (age, gender, city/state), academic (schooling, training) and professional information (exercise paid activity, length of profession, place of work) were collected from specialists and target audience.

In order to complement the analysis, evidence based on the response processes was investigated, to understand how people interpreted and interacted with the items and, thus, identify the possibility of the instrument being accepted and used as a support tool for decision making.

### Analysis of results and statistics

For data analysis, the CVI was calculated using the formula: number of responses “3” and “4” divided by the total number of responses^(19)^. Those that received a score of 1 or 2 were revised or eliminated. Item CVI > 0.80 and mean total and global CVI > 0.90 were considered excellent. The variables were categorized to perform the exact binomial distribution test for small samples, considering a significance level of 5% and a 0.80 proportion of agreement to estimate the statistical reliability of the CVIs.

The psychometric criteria were assessed by a minimum agreement of 80% among specialists (Table 1), in addition to cultural and relative aspects of content validity^(20, 21)^.

**Table 1:** Criteria for changing and deleting items, according to CVI and psychometric parameters.

	CVI	Psychometric parameters				
		Objectivity	Simplicity	Clarity	Precision	Relevance
Unmodified item	≥0.78	Agreement ≥ 80%	Agreement ≥ 80%	Agreement ≥ 80%	Agreement ≥ 80%	Agreement ≥ 80%
Modified item	≥0.78	Agreement < 80%	Agreement < 80%	Agreement < 80%	Agreement < 80%	Agreement < 80%
Deleted item	<0.78	Independent	Independent	Independent	Independent	Independent

*CVI: Content Validity Index*

As for the evidence based on the response processes, each item was read by the researcher during the interviews, so that the participants informed their understanding of what was asked and, thus, proceeded with the answer and possible suggestions. A written record was made of all the suggestions given by the participants regarding the elimination or better presentation of the items. In case of recurring doubts, the item has been reformulated or eliminated^(17)^.

## RESULTS

The construction of the items considered the pre-defined criteria and resulted in 105 in total (56 from the Subject Dimension and 49 from the Social Dimension). To validate their content, 25 experts were invited. Of these, 20 agreed to participate in the study, and 13 returned the completed instrument within the requested time. The 13 specialists who acted as judges were health professionals with proven professional experience, including physical education professionals and nurses; researchers with publications on the subject; and from seven Brazilian states (Ceará, Pernambuco, Amazonas, Piauí, Bahia, Rio de Janeiro, and Santa Catarina).

In all, 16 items were excluded because they were not considered representative of the latent trait studied, obtaining a CVI < 0.78. Of the 89 items that remained, those with ≤ 80% interexpert agreement on psychometric criteria were modified. Such modifications included substituting terms and words for greater clarity and simplicity. To ensure greater objectivity, precision and relevance, there was also the agglutination of items to form a single one, and the separation of items, to form more than one, resulting in a total of 101 at the end of the process.

An example is item 1 (Subject Dimension), which was changed from “What age group does your age include?”; items 26 and 27 (Subject Dimension), which were modified from “How much do you feel able to exercise in the presence of unfavorable physical sensations, such as tiredness, pain or discomfort?”; and item 29 (Social Dimension), modified from “In the neighborhood where you live and in the immediate vicinity, is there little availability of public services (health units/posts, post office, police station, airport)?” and “In the neighborhood where you live and in the immediate vicinity, are there few establishments that provide essential and non-essential services to the community (shopping centers, markets, banks, restaurants, pharmacy, religious temples, beauty centers, schools and the like)?”.

According to Chart 1 2, after these analyses, 101 items remained, with 84 modified due to the judgment of the psychometric criteria. Of these, 50 items were classified in the Subject Dimension; and 51, in the Social Dimension. The analysis of the binomial test shows that none of the items presented significant disagreements between the judges. The total CVI was 0.91 in the subject dimension; and 0.94, in the social dimension. The overall CVI, which considers the entire set of questions, was 0.92.

**Chart 1:** Content Validity Index and judgment of items in the Subject Dimension

Items - Subject Dimension		CVI	p Value*	JPC
1	Full age (in years)	1.0	0.055	< 80%
2	Birth sex?	0.84	0.502	< 80%
3	Race/Color?	1.0	0.055	< 80%
4	Current body mass (nutritional status)?	0.92	0.234	< 80%
5	In general, how would you rate your health?	1.0	0.055	≥ 80%^+^
6	Compared to other people of the same gender and age, how is your physical fitness?	0.92	0.234	< 80%
7	Has a healthcare professional told you that you have a chronic health condition: high blood pressure, diabetes, heart disease, asthma, neuromuscular conditions, cancer, or other?	0.84	0.502	< 80%
8	Do you feel weakness or lack of energy?	0.92	0.234	< 80%
9	Do you have physical-motor characteristics that limit your ability to engage in physical activity (arm/leg malfunction)?	0.92	0.234	< 80%
10	Do you have sensory characteristics that limit your ability to engage in physical activity (vision/hearing malfunction)?	0.92	0.234	< 80%
11	Do you have symptoms of depression?	1.0	0.055	< 80%
12	Do you have anxiety symptoms?	1.0	0.055	< 80%
13	Do you have frequent bouts of other mental disorders?	1.0	0.055	< 80%
14	Are you exposed to stressful situations that harm your well-being?	0.84	0.502	< 80%
15	Do you find your life discouraging?	1.0	0.055	< 80%
16	Are you bothered by the physical sensations experienced during exercise (sweating, labored breathing, rapid heartbeat)?	0.84	0.502	< 80%
17	Do you feel uncomfortable doing physical activities in front of other people?	1.0	0.055	≥ 80%^+^
18	Do you feel embarrassed about your body during physical activities?	0.92	0.234	> 80%^+^
19	Are you afraid of getting hurt while practicing physical activities?	0.84	0.502	< 80%
20	Do you feel motivated to practice physical activities?	1.0	0.234	≥ 80%^+^
21	Are you able to engage in physical activity if you are concerned?	1.0	0.055	< 80%
22	Do you feel able to practice physical activities if you are sad?	1.0	0.055	< 80%
23	Are you able to engage in physical activity if you are angry?	1.0	0.055	< 80%
24	Do you feel able to practice physical activities when the local conditions are bad?	0.92	0.234	< 80%
25	Do you feel able to practice physical activities when you are tired?	0.92	0.234	< 80%
26	Do you feel able to practice physical activities when you have body aches?	0.92	0.234	< 80%
27	Do you feel able to practice physical activities when you have no time?	1.0	0.055	< 80%
28	Do you feel able to practice physical activity when you don’t have support from another person?	1.0	0.055	< 80%
29	Do you like to practice physical activities?	1.0	0.055	≥ 80%^+^
30	Are you lazy to practice physical activities?	1.0	0.055	< 80%
31	Do you find physical activity boring or unpleasant?	0.92	0.234	< 80%
32	Do you have the self-control to stick with physical activity over time?	0.84	0.502	≥ 80%^+^
33	In everyday life, do you face situations that harm your health?	0.84	0.502	< 80%
34	Do you enjoy free or leisure time?	1.0	0.055	< 80%
35	In your free time, do you prefer to rest instead of doing physical activities?	0.92	0.234	< 80%
36	In the past, have you practiced any sport, dance, wrestling or other physical activity?	0.92	0.234	≥ 80%^+^
37	In your youth, did you use to participate in practical Physical Education classes?	0.84	0.502	< 80%
38	In your past, have you had serious injuries during the practice of physical activities that affect your interest in practicing them today?	0.84	0.502	< 80%
39	In your past, have you had any negative experiences during physical activities that affect your interest in doing them today?	0.84	0.502	< 80%
40	Do you eat well?	0.84	0.502	< 80%
41	Do you abstain from smoking?	0.84	0.502	< 80%
42	Do you abstain from alcoholic beverages or consume them in moderation?	0.84	0.502	< 80%
43	Do you sleep well?	0.84	0.502	< 80%
44	Highest level of education you completed?	0.92	0.234	< 80%
45	Do you believe that the regular practice of physical activities brings positive results for your health and well-being?	0.92	0.234	≥ 80%^+^
46	Do you know the effects of regular physical activity on health?	0.84	0.502	< 80%
47	Do you believe that the regular practice of physical activities helps in weight loss?	0.92	0.234	< 80%
48	Do you believe that the regular practice of physical activities helps in increasing the size of the muscles?	0.92	0.234	< 80%
49	Do you believe that the lack of physical activity can harm your health?	0.84	0.502	≥ 80%^+^
50	Do you have learning difficulties that interfere with the practice of physical activities?	0.84	0.498	< 80%

CVI: Content Validity Index; * p > 0.05 by binomial test for one sample; JPC: Judgment of psychometric criteria; + Unmodified item.

**Chart 2:** Content Validity Index and judgment of items in the Social Dimension

Items - Social Dimension		CVI	p Value*	JPC
1	Do you usually receive guidance from a physical education professional regarding the practice of physical activities for health?	1.0	0.055	> 80%^+^
2	Do your friends or family encourage you to practice physical activity?	1.0	0.055	< 80%
3	Do your friends or family invite you to practice physical activities?	1.0	0.055	< 80%
4	Are you responsible for looking after a small child in your family?	0.84	0.502	< 80%
5	Are you responsible for caring for a frail elderly person in your family?	0.84	0.502	< 80%
6	Are you responsible for caring for someone with special needs in your family?	0.92	0.234	< 80%
7	Do your family relationships present conflict or abuse?	0.92	0.234	< 80%
8	Do you experience situations of violence (assault, shouting, cursing, intimidation, harassment or similar)?	0.92	0.234	≥ 80%^+^
9	Do you experience situations of gender discrimination in places of leisure/practice of physical activities?	1.0	0.055	< 80%
10	Do you experience situations of ethnic-racial discrimination in places of leisure/practice of physical activities?	1.0	0.055	< 80%
11	Do you experience situations of religious discrimination in places of leisure/practice of physical activities?	1.0	0.055	< 80%
12	Main occupation?	0.92	0.234	< 80%
13	Is your income sufficient to meet your basic needs?	1.0	0.055	< 80%
14	Apart from what you spend on your basic needs, does your income have a reserve for personal and health emergencies?	1.0	0.055	< 80%
15	Do you receive aid or benefits (social and health) from the government to supplement your income?	0.92	0.234	< 80%
16	Have you and your family purchased transport goods (car or motorcycle)?	1.0	0.055	< 80%
17	Do you and your family hire domestic services?	1.0	0.055	< 80%
18	Does your home prevent you from doing physical activities because there is little space in it?	1.0	0.055	< 80%
19	Do you have materials to practice physical activities (clothes, shoes, bicycle, weights, or others)?	0.92	0.234	< 80%
20	Do you consider that your work/occupation demands a lot of physical effort?	0.84	0.234	< 80%
21	Do climatic factors in your region (rain, cold or heat) make it difficult for you to practice physical activity?	0.92	0.234	≥ 80%^+^
22	Near your home, do you find facilitators for walking/running on the streets (traffic lights, crosswalks, footbridges, signs or speed reducers)?	1.0	0.055	< 80%
23	Are there other residences close to yours (houses, buildings, or condos)?	0.84	0.502	< 80%
24	In your neighborhood and surroundings, do crimes occur (homicides, robberies, or drug trafficking)?	1.0	0.055	< 80%
25	Are there wild areas in your neighborhood and surroundings (unoccupied houses, stray animals, vandals, drunks or drug users)?	1.0	0.055	< 80%
26	In your neighborhood and surroundings, are the sidewalks and streets suitable for walking (flat, preserved and interconnected)?	1.0	0.055	< 80%
27	Is there public safety in places where you practice physical activity near your home?	1.0	0.055	≥ 80%^+^
28	Is there enough night lighting in the streets near your home and in places where you practice physical activities?	1.0	0.055	≥ 80%^+^
29	Is your home close to establishments that provide services to the community (shopping center, market, health center/unit, bank, restaurant, pharmacy, church, beauty salon, school or others)?	1.0	0.055	< 80%
30	In your neighborhood and surroundings, are there public places conducive to the practice of outdoor physical activities (squares, parks, beaches, gardens, lakes, trails, popular gyms, or exercise stations)?	1.0	0.055	< 80%
31	In your neighborhood and surroundings, are there public/private structures that encourage the practice of physical activity (walking paths, bike paths, sports courts, soccer fields or skate parks)?	1.0	0.055	< 80%
32	In your neighborhood and surroundings, are there private places conducive to the practice of physical activities (gym, fight, dance, swimming pool, club, active leisure center and similar establishments)?	0.92	0.234	≥ 80%^+^
33	Is your home close to places where you practice physical activities?	0.92	0.234	≥ 80%^+^
34	Near your home, do you see other people practicing physical activity?	0.92	0.234	< 80%
35	Does the neighborhood where you live, and its surroundings have air pollution, garbage or open sewage?	1.0	0.055	< 80%
36	Does the neighborhood where you live, and its surroundings have poorly maintained structures/no architecture?	1.0	0.055	< 80%
37	Are public physical activity facilities/equipment maintained for repair and upkeep?	1.0	0.055	< 80%
38	Do you see posters or information boards about the practice of physical activities in the places where you practice them?	0.84	0.502	< 80%
39	Nationality?	0.84	0.502	< 80%
40	Do your cultural differences limit your participation in physical activities performed in public/private places?	0.92	0.234	< 80%
41	Do you have access to essential public health services?	1.0	0.055	< 80%
42	Do you have access to private health services?	1.0	0.055	< 80%
43	Do you have free access to advice on physical activity?	0.92	0.234	< 80%
44	Do you have free access to guided physical activity practices?	0.92	0.234	< 80%
45	Do you have access to means of communication (internet, television, telephone, newspapers, or others)?	0.84	0.502	≥ 80%^+^
46	Do you face accessibility problems in public/collective spaces in your city?	0.84	0.502	< 80%
47	Do you enjoy the right to leisure where you live?	1.0	0.055	< 80%
48	Do you enjoy the right to the culture where you live?	1.0	0.055	< 80%
49	Do you enjoy the right to education where you live?	1.0	0.055	< 80%
50	Are there sports events or competitions in your city?	0.84	0.502	< 80%
51	Are there activities of labor gymnastics or other corporal practices in your work/study environment?	0.92	0.234	< 80%

CVI: Content Validity Index; * p > 0.05 by binomial test for one sample; JCP: Judgment of psychometric criteria; + Unmodified item.

The 101 items that had their content validated by experts were directed to validity based on response processes. This phase had the participation of 46 adults, with a mean age of 37.32 (+ 10.05) years; and minimum and maximum age of 18 and 58 years, respectively. People from all levels of education participated: 7 (15.2%) were only literate; 7 (15.2%) had only elementary education; 15 (32.6%), high school; 10 (21.7%), higher education; and 7 (15.2%), postgraduate.

People with different professions/occupations were included, such as teachers, lawyers, nurses, health agents, commercial workers, students, day laborers, farmers, painters, drivers, caregivers of the elderly and children, as well as unemployed people. In all, the majority were female (58.7%), brown (56.5%), single (43.5%), had an income of up to one minimum wage (60.9%) and practiced physical activity during leisure (56.5%).

The evaluation of the items based on the response processes, in the form of an individual interview, revealed that 35 of them needed changes to become more understandable, especially for the lower strata of the population (Chart 3). In some items, few word or term changes were made (7, 21, 22, 23, 27, 34, 37, 44, 46 – Subject Dimension; 1, 4, 5, 6, 20, 26, 27, 30, 31, 32 – Social Dimension); in others, the structure of the question was changed (6, 9, 10, 41, 42 – Subject Dimension; 18, 23, 25, 47, 48, 49 – Social Dimension).

**Chart 3:** Items from the assessment of vulnerability to physical inactivity changed after evaluating the response processes: Subject and Social Dimension

Item	Items with changes after response processes
Subject Dimension	
6	In general, how would you rate your ability to perform daily physical activities?
7	Have health professionals told you that you have a long-term health problem (high blood pressure, diabetes, asthma, heart problems, cancer...)?
9	Do you have trouble moving or moving any part of your body?
10	Do you have any vision/hearing impairments that affect your ability to exercise?
21	Do you feel able to engage in physical activity when you are worried?
22	Do you feel able to practice physical activities when you are sad?
23	Do you feel able to practice physical activities when you are angry?
27	Do you feel able to practice physical activities when you have little time available?
34	Do you enjoy free or leisure time on a daily basis?
37	During the school period, did you participate in practical Physical Education classes?
41	You smoke?
42	Do you use alcoholic beverages?
44	Education (complete)?
46	Are you aware of the effects of regular physical activity on health?
Social Dimension	
1	Do you receive guidance from a physical education professional?
4	Do you take care of a small child in your family?
5	Do you take care of a frail elderly person in your family?
6	Do you care for someone with special needs in your family?
18	Does the physical space of your home make it difficult to practice physical activities at home?
20	Do you consider that your work/occupation requires a lot of physical effort?
23	Are there other occupied residences close to your home?
25	In your neighborhood and surroundings, do you often encounter drug users, vandals, or drunks?
26	Do you consider that the sidewalks and streets in your neighborhood are suitable for walking (flat, preserved and interconnected)?
27	Is there security/policing at places where you practice physical activity near your home?
30	In your neighborhood and surroundings, do you have access to public places conducive to the practice of outdoor physical activities (squares, parks, beaches, gardens, lakes or trails)?
31	In your neighborhood and surroundings, do you have access to public/private structures that encourage the practice of physical activity (walking paths, sports courts, soccer fields, popular gyms, bike paths, exercise stations or skate parks)?
32	In your neighborhood and surroundings, do you find private places conducive to the practice of physical activities (gym, fight, dance, swimming pool, club, active leisure center or similar establishments)?
47	Do you have access to leisure activities in the region where you live?
48	Do you have access to cultural activities in the region where you live?
49	Do you have access to public education in the region where you live?

In addition, some items had to be eliminated due to their inadequacy and lack of understanding by the population, even after some adjustments and explanations (50 – Subject Dimension; 36 – Social Dimension) or because they were considered redundant in the face of the set of items (38 – Dimension Subject; 33 Social Dimension).

*I think these two questions* [38 and 39] *refer to the same thing. Because having a serious injury is already a negative experience. I could just leave 39, because it already includes all the possibilities.* (Person 39)

*You ask if my house is close to these places* [33]*, but you asked before if my neighborhood had a square, park, gym, court* [30, 31, 32] [...] *I think it’s the same answer, because if it’s in my neighborhood, it’s close to my house.* (Pessoa 32)

*Poorly preserved structure* [36]*, it depends on the people, right? Sometimes yes, sometimes no. It depends on a lot* [...] *There are beautiful things and some ugly things* [...] *And this architecture, what is it? Are those tall buildings?* (Person 03)

*I didn’t understand what these learning difficulties would be.* [50] *Learning what?* (Person 08)

*Some people may not understand the meaning of the term* [50]. *Furthermore, this question is not able to indicate the presence of a decrease in cognition or intellectual disability.* (Person 45).

*In general, the set of items presents important questions to understand vulnerabilities related to physical inactivity. Some well-established in the literature and others not yet, but that need visibility.* (Person 42)

In the end, the entire process resulted in the permanence of 97 items (with 48 in the Subject Dimension and 49 in the Social Dimension), with evidence of content validation and based on the response processes. Therefore, these were considered valid for the representation of the studied latent trait.

## DISCUSSION

The present study presents a database with 97 items for measuring vulnerability to physical inactivity in adults, organized into two dimensions (Subject and Social), with appropriate parameters and validity evidence.

In general, the validation process is a judgment about the congruence between the latent trait and its physical representation. It refers to a measure based on the evaluation of the subjects and content addressed in each item of an instrument^(20)^. This, when properly developed, can influence decisions about care, interventions, and policies. To do so, it draws on the relevant literature, the researcher’s experience, the opinion of specialists in the area, content analysis, state of the art and characteristics of the context^(22)^.

The construction of these evaluated items was based on theoretical reflection on issues of vulnerabilities and inequities in health and on the elucidation of the dimensionality and the constitutive elements of vulnerability to physical inactivity^(12)^. Such reflections were anchored in evidence from national and international studies, obtained through a scope review about intervening factors in physical inactivity, whose protocol is registered in an open platform^(23)^. This process was complemented by the knowledge of specialists working in different regions, to ensure the coverage of aspects and language that consider Brazilian cultural diversity. These evaluated the representativeness of the studied latent trait and produced evidence of validity of its content.

In this process, some items were excluded because they did not reach a consensus among experts regarding their representativeness in the latent trait. As for the judgment of the psychometric criteria, most of the items that remained underwent changes to meet the criteria to which they were submitted (objectivity, simplicity, clarity, precision, relevance).

The reflection of the behavioral paradigm was noted, as most of the validated items refer to individual aspects, which touch biological, psychological, and cognitive factors. However, a social understanding of the phenomenon also emerged, as some items about the economic, political, and environmental context were also validated.

This fact is linked to discussions that centralize new contexts of thought supported by social paradigms present in the current situation. Much has been discussed about the influence of social inequalities on adherence to body practices and physical activities, in which scenario some population groups are at a notable disadvantage^(8)^. Such inequalities are established in several health indicators, especially when innovations appear in actions to promote leisure physical activities, which tend to reach the most socially and economically privileged^(9)^.

There are several factors that influence physical inactivity, such as age, sex, race/color^(24)^, physical environment, social support, physical situation^(25)^, personal motivation^(26)^, risk behaviors^(27)^, in addition to factors such as immigration^(28)^, culture^(29)^, violence and alcohol abuse^(30)^, work situation and social isolation^(3)^.

Given this evidence, the items presented include a range of these variables and can be understood in a two-dimensional format (Subject and Social), by recognizing the dynamic and inter-relational character of the constituent elements of the subject’s vulnerability and social vulnerability. The Subject Dimension refers to the elements formed based on intersubjective relationships, in which the freedom of tension between knowledge and power weighs, which makes self-recreation possible. The Social Dimension refers to what the different means of interaction of the subject with others presuppose, based on the scenario in which the recognition and expression of the being is possible^(11)^.

Among the various existing approaches^(31,32)^, vulnerability analysis refers to the way in which individual aspects interact with cultural and social dynamics to produce conditions that increase the chances of threats and dangers materializing^(10)^. By considering the negative impact of physical inactivity on the health of populations^(4,5)^, the aim is to understand how the connection between the various personal and contextual aspects can increase the chances of physical inactivity at an individual and collective level.

Faced with the complexity of interactions involving physical inactivity, it is necessary that, in addition to behavioral aspects, aspects of vulnerability also be considered, since it is recognized that certain unfavorable individual, environmental and social conditions, combined with the fragility in facing these difficulties, limit the choices of people and make them more susceptible to physical inactivity^(12)^.

Such vulnerability conditions need to be considered when assessing the situation of populations for effective planning and development of actions to combat physical inactivity. This applies, especially, to multidisciplinary strategies developed in health services, for populations with or without non-communicable communicable chronic diseases^(33)^. In addition, such elements help in better understanding, review, application, and improvement of the nursing diagnosis “Sedentary Lifestyle”, included in the North American Nursing Diagnosis Association, in 2004^(34)^.

Evidence of validity based on the response processes could ensure the comprehensibility of the items for the lower strata of the population and avoid inappropriate language. Of the changed items, most underwent replacement of words or terms. Some had their structure modified to present an idea with simpler and more unambiguous expressions.

Despite adjustments and explanations, some items had to be eliminated at this stage. As evidenced in the participants’ speeches, the lack of understanding of the lower strata was due to lack of knowledge of complex concepts and aspects, such as “architecture” and “learning”. However, there was no damage to the item’s content, as these aspects were evaluated in a general way in other items in the set, without compromising the breadth criterion. The same occurred with the items considered redundant in the face of their set, that is, with those that had aspects already covered in other items. In general, the changes made were essential to avoid measurement biases and to make the set of items clear and easy to understand, without compromising idiomatic, cultural, and semantic equivalences.

It should be noted that this entire evaluation process permeates the development of valid and reliable measurement instruments. Validated instruments are important not only for health assessment, but for scientific research and professional practice in the most distinct areas of knowledge. In this sense, validation studies help researchers and professionals to choose the best tool to ensure the quality of the results obtained^(35)^.

### Study limitations

This study presents as a limitation the generalized analysis of the results, caused by the high number of items and specificity of the psychometric criteria evaluated.

### Contributions to the Area

The aspects addressed in this study, in addition to helping to improve the nursing diagnosis “Sedentary Lifestyle”, serve as a basis for studies aimed at building validated measurement instruments, which can provide important indicators for the care of patients according to their needs. In addition, the present work can equip physical education professionals, nurses and other health professionals, in a multidisciplinary approach, in the planning of interventions to promote physical activity for the health of populations in different contexts.

## CONCLUSION

By understanding physical inactivity as an impediment to the healthy development of people and populations, vulnerability to physical inactivity involves aspects that have a negative impact on the biological, physical, social, and emotional dimensions of human life. A better understanding of the phenomenon requires the development of its measurement.

The items presented have adequate validity and appearance parameters and can subsidize the construction of evaluative instruments, which consider the subject’s vulnerability and social vulnerability. It should be noted that this study refers to the initial stages of the construction process of a measurement instrument and that it is still necessary to carry out additional analyzes for the safe use of an instrument in the diagnosis of the population, allowing the elaboration of strategies to face physical inactivity.
